# Physiology, Pathology and Regeneration of Salivary Glands

**DOI:** 10.3390/cells8090976

**Published:** 2019-08-26

**Authors:** Cristina Porcheri, Thimios A. Mitsiadis

**Affiliations:** University of Zurich, Institute of Oral Biology, Plattenstrasse 11, CH-8032 Zurich, Switzerland

**Keywords:** salivary glands, oral epithelium, xerostomia, exocrine glands, salivary gland-resident stem cells

## Abstract

Salivary glands are essential structures in the oral cavity. A variety of diseases, such as cancer, autoimmune diseases, infections and physical traumas, can alter the functionality of these glands, greatly impacting the quality of life of patients. To date, no definitive therapeutic approach can compensate the impairment of salivary glands, and treatment are purely symptomatic. Understanding the cellular and molecular control of salivary glands function is, therefore, highly relevant for therapeutic purposes. In this review, we provide a starting platform for future studies in basic biology and clinical research, reporting classical ideas on salivary gland physiology and recently developed technology to guide regeneration, reconstruction and substitution of the functional organs.

## 1. Introduction

Structures secreting fluid to facilitate feeding emerge progressively throughout evolution and can be found in very simple organisms (e.g., *Caenorabditis elegans*) and more complex species (e.g., *Drosophila melanogaster*, placental mammals). In humans, major and minor salivary glands produce and secrete digestive fluids or protein-rich fluids. The three pairs of major salivary glands (i.e., parotid, submandibular and sublingual glands) are responsible for the production and secretion of saliva in the oral cavity, whose moisturizing effect preserves oral hygiene and allows taste, speech and mastication [1].

The parotid gland (PG) is mainly composed of serous acini-secreting α-amylase-rich saliva [2]. The sublingual gland (SL) secretes mucous, a viscous solution rich in mucins [3,4,5]. The submandibular gland (SMG) is composed by a mixed population of acini with a mucous and serous function [1,4,6]. These three major salivary glands account for more than 90% of salivary secretion. Minor salivary glands are distributed throughout the oral cavity, specifically in the labial and lingual mucosa, as well as palate and floor of the mouth.

Saliva is an essential fluid for oral cavity maintenance and functionality. Digestive enzymes within saliva initiate the digestion process, and at the same time, saliva acts as a lubricant of solid nutrition, thus helping its passage through the esophagus. By moisturizing the tongue and other tissues of the oral cavity, saliva has an essential role in speech and taste sensitivity [7]. It also balances the pH of the mouth, thus protecting the soft oral tissues and teeth from an extended exposure to an acidic environment. Saliva contains several signalling molecules, such as EGF, FGF, NGF and TGF-α, that are essential for the regeneration of oral and oesophageal mucosa. Finally, the antibacterial and antifungal components of the saliva, such as lysozymes, immunoglobulins and lactoferrin, inhibit the progression of bacterial infection and dental caries.

Physiological functions and the histological appearance of salivary glands are rather conserved between species and individuals, but clear distinctions exist in terms of anatomical position and volume (i.e., the PG is the largest of the salivary glands in humans, while the SMG is the largest in mice). In humans, 20% of individuals possess an accessory PG, equipped with its own blood supply, and a secondary excretory duct independent from the main body of the gland [2]. A relevant sexual dimorphism appears in mice: SMG glands differ significantly between male and female mice, in particular the granular convoluted tubules (GCT), which are larger and more numerous in the male organs. Such a dimorphism has been lost in humans, and GCTs are absent in human SMGs, independently of gender [8]. Murine submandibular glands specifically synthetize EGF and NGF in the GCTs following testosterone activity, while their production in humans occurs in the striated ducts. These differences reveal an interesting species-specific evolutionary tract in oral morphology and ductal specification [9].

## 2. Salivary Gland Anatomy and Morphogenesis

### 2.1. Morphogenesis of the Salivary Glands

**Figure 1:**
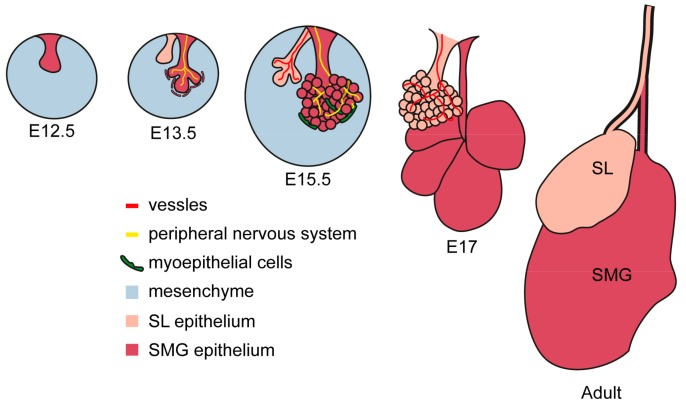
Embryonic development of murine SMG and SL glands. Embryonic development starts with an epithelial placode, which, in turn, invades the mesenchymal layer and branches up. Innervation and vascularization advance in parallel with the growth of salivary glands primordia and directly regulates its maturation. Myoepithelial cells arise from the external epithelial layer and develop into the contracting unit involved in the regulation of secretion.

Salivary glands originate from an epithelial placode during embryonic development (from E11 to E16 in mice and between the 4th and the 12th embryonic weeks in humans). The initial placode grows and extends into the underlying mesenchyme, acquiring a bud shape. The growing epithelial bud progressively stratifies with concentric layers each formed by a specialized cell type. During branching morphogenesis, the initial salivary bud divides into additional, independent buds that grow and cleave again, until the formation of an extensive arborization typical for the mature salivary gland (Figure 1) [10].

A portion of the cells forming the outer epithelial layer of the buds differentiates into myoepithelial cells. They will then acquire smooth muscle characteristics and locate in direct contact with the acinar structure to regulate the release of secretion [11]. Inner epithelial cells differentiate further to acquire a distal (tips) or proximal (stalk) identity, which, in turn, evolves into acini or ducts, respectively [12].

Both epithelial and mesenchymal cells produce the basement membrane and stromal extracellular matrix. The composition of the extracellular matrix varies from region to region during branching, and bundles of collagen I, IV and fibronectin are thought to directly control the maturation process. Low concentrations of fibronectin and glycosaminoglycans facilitate the area of bud growth, while accumulation of fibronectin, collagen IV and glycosaminoglycans limit epithelial activity of the peripheral nervous system also participates in directing salivary growth and stabilizing the basal lamina, determining sites where branching and clefting occurs (Figure 1) [13,14]. The peripheral nervous system participates in directing salivary gland maturation. The primordial epithelial structure is innervated by cholinergic neurons, whose axonal growth follows the ramified pattern of the developing gland [15]. Local release of acetylcholine induces proliferation of epithelial progenitor which positively regulate epithelial branching [16,17,18].

A network of capillaries derived from terminal arterioles develops in parallel with the acini-duct systems and has an instructive role in the establishment of the epithelial patterning [19].

### 2.2. Histological and Anatomical Features

The three major salivary glands have a similar anatomical structure, with a main secretory duct extending from the main body of the gland to the oral cavity. The secretory duct of the SMG is the Wharton’s duct, which reaches the oral cavity under the tongue at the sublingual caruncula. The Bartholin duct is the major duct of the SL and it connects with the Warthon’s duct at its extremity before the opening in the oral mucosa. SL also have smaller ducts called Rivinus’s ducts that release secretion beneath the tongue onto the floor of the mouth. Finally, the PG has an independent duct called Stensen’s duct, opening on the upper portion of the oral cavity.

**Figure 2:**
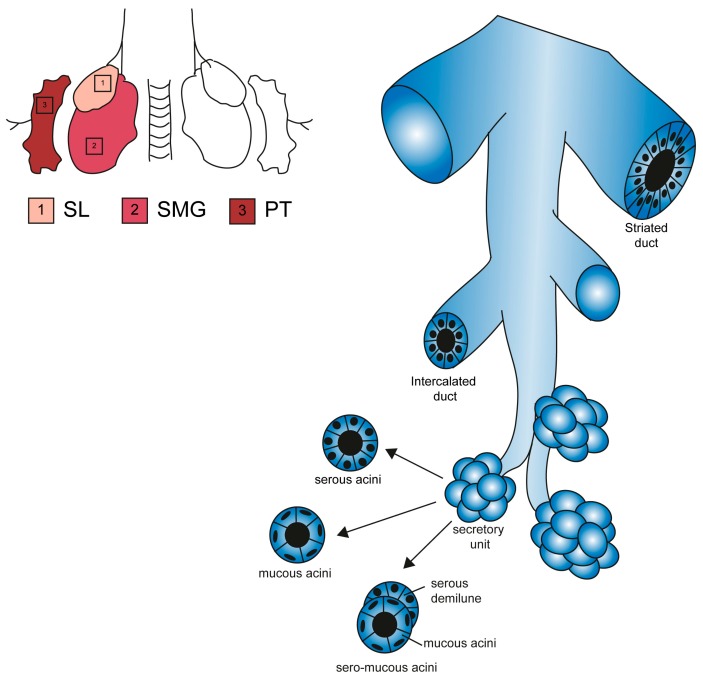
Structural features of major salivary glands. Schematic representation of SL, SMG and PG glands and their functional elements [35]. All glands contain ducts of bigger diameter (striated duct) and branching ducts (intercalated ducts). The acinar end-pieces are secretory units specialized in a single type of secretion (serous, mucous or mixed).

The secretory duct branches up into striated ducts, composed of columnar epithelial cells whose appearance is due to infoldings of the basal membrane. Striated ducts extend further into progressively smaller intercalated duct, characterized by a wall of flat cuboidal epithelial cells. Finally, the structure ends into a secretory unit of acinar cells grouped as end-pieces specialized in producing and releasing the primary secretion (Figure 2).

### 2.3. Innervation and Trophic Support

Salivary glands are densely innervated by the autonomic nervous system.

The parasympathetic nerves release acetylcholine, which activates the muscarinic receptors stimulating fluid secretion. The sympathetic nerves, on the other hand, control salivation through release of noradrenaline and activation of α-and β-adrenoreceptors, the first stimulating fluid-rich secretion and the latter protein-rich secretion [20,21]. This suggests that the serous population of cells is innervated by the parasympathetic system, while the mucous population mainly depends on the sympathetic stimulation [22,23,24]. The distribution of the secretory nerves highly depends on species, age and type of gland [25].

Innervation of the salivary glands starts during embryonic development and progress in parallel with the organ definition. Neural crest-derived cells migrate to their appropriate location in the oral epithelium to instruct the thickening for the placode formation. The neural crest-derived precursors differentiate to form the parasympathetic submandibular ganglion (PSG) surrounding the epithelial primordia of the major secretory duct. As branching proceeds further with the developmental process, axons from the PSG extend along the epithelium to envelop the secretory end-pieces. By E14 in the mouse, the gland is highly branched and fully innervated [26]. The instructive role of the PSG is currently gathering interest, as ablation of the PSG reduces expression of epithelial progenitor markers such as Krt5 and Krt15, and might, therefore, be implicated in the maintenance of endogenous stem cells [27]. Similarly, acetylcholine induces proliferation of Krt5-expressing progenitor cells via regulation of the EGF pathway, suggesting that the parasympathetic activity coordinates the maintenance of the undifferentiated pool and their balance during organogenesis [27].

The development of the sympathetic innervation proceeds conjointly with acinar and ductal maturation, which suggests a role in the final specification of the salivary gland.

The primary sympathetic salivary centres are located in the upper thoracic spinal cord, and reach the salivary gland via the superior cervical ganglion. Sympathetic axons enter the SMG in parallel with the vascular system, and vascular-derived guidance cues are needed for sympathetic neurons to grow. Mice lacking endothelin3 or endothelin-receptor type-A have reduced sympathetic innervation of the salivary glands and defects in SMG secretion [28,29]. 

Despite developing with a similar timing and being surrounded by the same mesenchymal cap, the SL gland contains only a few sympathetic nerves, while the SMG has rich innervation. This dichotomy is probably to be associated with the different levels of NGF (high in the SMG and low in SL) [30]. After submandibular gland removal, the NGF levels drop dramatically in the plasma, to then go back to a normal level after several weeks. It has, therefore, been postulated that the submandibular gland in mice might work as source of NGF [31]. Nevertheless, removal of the SMG in mice has no deleterious systemic effect, indicating more of an accessory role in NGF secretion rather than a primary one [32].

Once reaching the salivary gland, the independent innervation of parasympathetic and sympathetic efference bundles up together surrounded by Schwann myelinating cells [33]. Dual innervation can be found in myoepithelial cells, acinar end-pieces and local blood vessels, all of which plays a functional role in salivary gland secretion [34].

## 3. Chemistry of Secretion and Functions of Salivary Glands

Secretion itself is a combination of three events: (i) nervous cholinergic stimulation initiates fluid filtration from blood plasma to the acinar lumen, (ii) exocytosis of cytoplasmic granuli-containing proteins into the acinar lumen, and (iii) mechanical contraction of the secretory end-pieces mediated by specialized myoepithelial cells. 

### 3.1. Chemical Composition of Saliva

**Figure 3:**
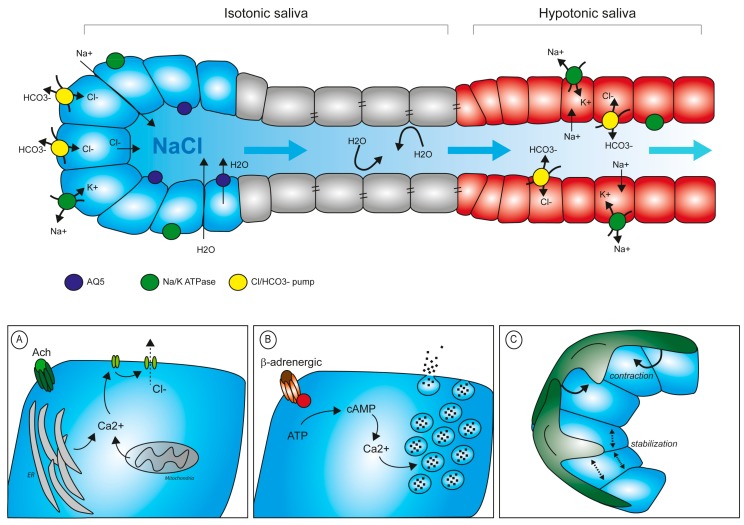
Saliva production and regulation of secretion. Composition of primary saliva and its progressive conversion into a hypotonic fluid. Primary saliva is rich in electrolytes which attract water into the lumen. In a secondary step, electrolytes are resorbed via the epithelial duct cells to reduce their concentration in the saliva before release in the oral cavity. (**A**) Schematic representation of intracellular Ca^2+^ control levels upon muscarinic stimuli. (**B**) Secretion of proteins into the lumen requires Ca^2+^-mediated degranulation upon β-adrenergic stimulation. (**C**) Myoepithelial cells participate in the mechanical contraction stimulating secretion and in the stabilization of acinar structure during the resting phase.

Primary saliva is initially produced and secreted in acinar cells. Epithelial cells of serous end-pieces are sealed between them via tight junction to preserve apical-basal polarity. This guarantees that electrolytes do not pass freely in between the cells, but require a system of ion-pumps and channels to finely regulate the intracellular and extracellular concentration of each electrolyte. Specifically, the Na/K ATPase pumps sodium out of the cell, which then accumulates in the intercellular space. Upon cholinergic stimulus, Ca^2+^ is released from intracellular storage sites (i.e., ER, mitochondria) and its concentration increases of 5–10-fold in the cytoplasm. This chemical change, in turn, opens the channels for Cl^−^, which diffuses into the acinar lumen following its concentration gradient. Na^+^ will then also translocate through the paracellular space to the lumen. Water from the adjacent capillaries will pass into the lumen by osmosis, both through paracellular flow and specialized aquaporin channels (mainly AQP5) [36]. The thus-formed primary saliva accumulates in the acinar lumen and starts diffusing along the secretory ducti. During its passage in the ducts, the chemical composition of saliva is significantly modified. Cells of the striatal ducts contain Na/K ATPase pumps and carbonic/Cl pumps to extract NaCl from the primary saliva and reduce salt concentration in the fluid, while an increased number of tight junctions keeps ductal cells sealed, avoiding water loss (Figure 3). The final serous secretion is, therefore, a watery hypotonic fluid.

### 3.2. Protein Components of Saliva and Degranulation

Saliva also contains an important mixture of proteins, mainly proteolytic enzymes and mucins. These products are synthesized by acinar cells and stored in cytoplasmic granules. Each major salivary gland produces a specific proportion of protein type, defining a unique composition of saliva per type of gland.

Similarly to synaptic degranulation, the salivary granules are released into the lumen following several steps of exocytosis: docking, priming, fusion and removal [37]. The molecular regulation at the basis of these events is largely unexplored, but evidence points towards interactions between specific ligand-receptor (SNAP-SNAREs molecules) for the initial contact of the granule with the plasma membrane [38]. Priming prepares the granules for its future structural modification before entering the phase of fusion, in which the content of the granule is released into the acinar lumen. 

The whole cycle is rather fast and is completed within 30 min of initial stimulation. In serous and seromucous glands, sympathetic nerve stimulation activates β-adrenoreceptor, which, in turn, increases intracellular levels of cAMP and ultimately leads to degranulation [39]. In PG and SMG glands, release of granule contents induces shrinkage of the single acinar cell, which visibly reduces in size after degranulation [40]. The SL gland is mainly secreting mucous, a fluid rich in mucins, high molecular weight glycoproteins composed by 66% of carbohydrate, and characterized by a high content in serine and threonine [41].

Salivary mucins differ from other mucins of the body (i.e., intestinal mucins) by their high content in sialic acid, which confers pH neutrality and makes them unreactive to classical alcian blue staining. The high amount of mucin produced by these glands correlates with the high proportion of mucous acini cells. Exocytosis of mucous granules importantly differs from serous or seromucous events [42]. In sublingual mucous acini, exocytosis is mainly driven by muscarinic stimulation and does not occur simultaneously for all granules of acinar cells. Although the dynamic of salivary gland secretion needs further clarification, current advances in confocal and live-imaging technology will certainly help the understanding of the salivary secretion in the near future [43].

The SMG gland produces both serous and mucous products. The structure of the mixed secretory end-pieces shows a line of serous acini juxtaposed on the mucous acini structure (Figure 2). The number of mucous versus serous acini is not equal in the gland, and the majority of the secretion in the SMG is mucous. Mucin levels are 10-fold higher in the SL compared to the SMG, while mucins are undetectable in the PG [41]. Additionally, the mucins produced by the two mucous or seromucous glands are chemically different—nearly all sialic-acid is mucin-bound in the SL, while only 40% of sialic residues were found bound to mucins in the SMG [41]. These observations suggest that each gland is responsible for a unique type of secretion most probably underlining a distinct function in the oral cavity [41,44].

One of the most important proteolytic enzymes secreted with saliva is α-amylase. This enzyme is essential for breaking down starch into maltose, a disaccharide fundamental for our diet. α-amylase activity is mainly found in PG secretions and much reduced (1000-fold less) in saliva produced by SL and SMG [44].

Another important protein product of salivary glands is lysozyme, a proteolytic enzyme with antimicrobial functions. Recent findings associate lysozyme activity with membrane permeabilization on both Gram-positive and Gram-negative bacteria, as well as in fungi [45,46]. Additionally, lysozyme also has anti-viral properties and plays a role in inducing tumour cell lysis [47,48]. These novel roles identify lysozyme as a major player in clearing the oral mucosa from a variety of biological threats and preserving homeostasis of the oral cavity. The lysozyme, is mainly produced by the demilune portion of the SMG and SL, while in the PG production of lysozyme is very limited [49,50].

### 3.3. Mechanical Control of Saliva Secretion

Sympathetic and parasympathetic stimuli can act directly on the myoepithelial cells embracing the acini end-pieces to initiate contraction. Myoepithelial cells contract in repose to nerve stimuli transferring a mechanical pressure to the acinar cells, which contributes to degranulation and saliva movement. Additionally, myoepithelial cells stabilize the structure of the acini, avoiding back-pressure stress after secretion and back diffusion of saliva upon external mechanical compression [51]. When myoepithelial functionality is experimentally blocked by α-adrenoceptor inhibition, secretion still occurs but the fluid movement is dramatically slowed down [52].

## 4. Salivary Gland Disorders

### 4.1. Tumours

Tumours originating in salivary gland tissue are often benign. In minor salivary glands, the most common clinical complication is the formation of mucus retention cysts, a non-malignant evolution of the altered tissue [53].

Major salivary glands mainly display epithelial malignancies (carcinomas), with very heterogeneous features and occasional neuroendocrine differentiation. Malignant salivary gland tumours represent about 5% of all head and neck cancers, with a slight predominance in men [54,55].

The aetiology of the salivary gland tumour is mainly described by the multicellular theory, by which each cell type can give rise to a specific type of tumour (Table 1) [56,57,58,59,60].

In addition to being a site for primary tumours, salivary glands are also a site for tumorigenic cells to establish metastases, originating from other primary cancers, mainly skin malignancies.

The most common malignant tumour is mucoepidermoid carcinoma, potentially arising in any salivary tissue, but mostly affecting the PG. Mucoepidermoid carcinoma is associated with a specific genetic translocation between chromosomes 11 and 19, which produces a fusion gene (*MECT1*) involved in the Notch-pathway and cAMP-responsive element binding (CREB) activation. The product of gene fusion was detected in genomic screenings together with alteration of the EGF-receptor pathway and aberrant activation of p53 regulator of apoptosis [61,62]. The specificity of the novel gene-fusion for mucoepidermoid carcinoma cells has improved diagnostic capacity and brought increased focus on the study of the molecular mechanisms supporting the disease. Identification of the fusion product (CRTC1-MAML2) or constitutive activation of the Notch pathway via high levels of the Notch target *Hes1* are indicative of cancer progression [63,64].

Similarly, in adenoid cystic carcinoma, mutations in *NOTCH1* and *NOTCH2* have been identified as altered targets in various genomic screenings [65,66], and knock-down of *Notch1* or *Notch2* inhibits proliferation of adenoid cystic carcinomas in models of the disease [67,68]. These findings suggest that this pathway has a central role in the initiation of the most common neoplasms affecting salivary glands.

### 4.2. Primary Sjögren’s Syndrome

Primary Sjögren’s syndrome (pSS) is a systemic autoimmune disease affecting salivary and lacrimal glands. Often accompanying other immune system disorders (such as lupus and rheumatoid arthritis), its main effect is the loss of mucous membrane and moisture-secreting gland cells, resulting in xerostomia and xerophthalmia. Although the pathogenesis of the disease remains largely unknown, the role of the B-lymphocytes appears to be essential in the initiation of the disease. Members of the TNF superfamily (such as BAFF/APRIL) are produced not only by patrolling immune cells but also by the epithelial cells of the salivary glands. Through these pathways, B-cells are activated and start to proliferate in an uncontrolled manner [69,70,71]. Their pivotal role includes infiltration of the salivary glands to produce an ectopic germinal centre and local secretion of autoantibodies. The centre can grow independently from the surrounding tissue and can evolve in more complex diseases such as non-Hodgkin lymphoma [71].

To date, there is no efficient treatment available for pSS, and symptoms may only be attenuated. Specific antibodies for BAFF (Belimumab) show limited relief [72], but combination with other immune-therapies might prove to be more efficient [73]. Promising approaches using monoclonal antibodies anti-CD20 (Rituximab) or anti-CD22 (Epratuzumab), are currently under examination [73,74,75,76].

### 4.3. Post-Irradiation Syndrome

Radiotherapy is an important main or complementary treatment in a variety of cancers, including head and neck tumours. One of the most significant side effects of local irradiation is an alteration of salivary glands functionality, resulting in hyposalivation and xerostomia [77]. Exposure to radioactive sources causes DNA damage, leading to cell death or cell senescence in proliferating cells. Specifically, irradiated salivary glands rapidly lose acinar cells, with dramatic functional impairment [78]. Hyposalivation results in chronic dryness of the oral cavity, which, in turn, leads to ulceration, infections, increase exposure to caries, periodontal diseases and hampered speech and mastication [79]. The only treatment available to cope with xerostomia is the topic application of substituting agents, such as saliva substitute and mucosa lubricant [80,81]. Pharmacologically, pilocarpine and cevimeline administration are systemic drugs for the treatment of dry-mouth conditions, but their efficiency requires the presence of functional tissue. All therapies currently available for the treatment of xerostomia provide only temporary relief, and require multiple applications for a long period of time [82].

### 4.4. Infections

Several viruses and bacteria infect the tissue of salivary glands in a specific manner. Endemic parotitis is due to infection of the mumps virus and lead to PG swelling and systemic symptoms. It is mainly affecting children in pre-scholar age and treatment is primarily symptomatic. The HIV virus can infect the PG and induce the formation of cystic lesions with surgical resection being the most common treatment procedure. Hepatitis C and coxsackievirus are RNA-bound viruses, able to infect salivary glands and damage the host tissue, leading to xerostomia. One of the main routes of viral spreading is the gland secretion itself and thus transmission through saliva exchange is the major infection mode.

Bacterial infection is very rare and mainly affects the PG in patients already debilitated by other conditions, such as diabetes, recovery after surgery or immunodeficiency. Therapeutic treatments reducing saliva flow help the establishment of bacterial colonies in the mucosa and increase the risk of infection, mainly from *Streptococcus* strains and *Staphylococcus aureus*. Mycobacter infection is more common in infants where it locally grows masses that might break the skin of the patient leaving scarring of the tissue. Non-controlled bacterial infection might spread beyond the gland borders and invade the deep space of the neck with possible serious complications including septicaemia. Chronic inflammation might also lead to sialadenitis, an accumulation of lymphocyte infiltrate in the duct system with consequent obstruction and hampering of the secretory system. Clinically, this results in xerostomia and local painful swelling [83].

## 5. Molecular Pathways Involved in Salivary Gland Functionality

A complex network of molecular pathways ensures full functionality of salivary glands.

Within the growth factor family, the FGF pathway is a major player in salivary gland homeostasis. Both intercalated and excretory duct cells express FGF-receptor2IIIb, while FGF7 can be found in salisphere-forming cells, suggesting a preserved trophic role [84]. The Wnt pathway is an essential pathway during embryonic organogenesis and it participates in salivary gland maturation at two distinct time points: during mesenchymal activation and later in the ductal epithelium differentiation and formation of the lumen [85]. In adult salivary glands, expression of the Wnt signalling is maintained in the duct epithelium and its activity is promoted during regeneration post-injury or expansion of progenitor cells [86]. Hedgehog (Hh) signalling is activated during branching morphogenesis and it is impaired upon blockage of the ligand in vivo and in vitro [87]. During functional regeneration, the Hh-target gene *Gli1* promotes epithelial proliferation, and overexpression of the pathway has been recently associated with the reactivation of a pool of salivary gland progenitors [88].

In *Drosophila*, The Notch pathway also plays a role during development in the determination of salivary ducts [89]. Receptors NOTCH1 and NOTCH4, the ligands JAGGED1, JAGGED2 and DELTA1, together with the target gene *Hes1*, are expressed in adult murine salivary glands and are associated to regulation of differentiation and growth [90]. Particularly, Notch signalling is activated during regeneration after duct injury, suggesting that Notch plays a pivotal role in the maintenance of the tissue homeostasis and regeneration, although its role in physiological conditions needs further clarifications [91]. Adenocarcinoma cells are often associated with accumulation and uncontrolled proliferation of immature ductal cells, and are associated with hyperactivation of Notch (Table 1) [92].

Since embryonic development, innervation plays an important instructive role in the functionality of the salivary gland. Parasympathetic nerves mainly rely on the neurotrophin GDNF for their development, and genetic deletion of *Gdnf* results in reduced number of innervated acini in the SL [93]. On the other hand, the sympathetic innervation mainly depends on NGF. In agreement with its high concentration, NGF was initially isolated from the murine SMG [94]. NGF signals through two receptors (high and low affinity) widely distributed on cells in the soft tissues of the oral cavity, to regulate cell survival and axonal growth [95]. In particular both acinar and ductal cells express NGF receptors, and they coordinate wound healing, angiogenesis and tissue remodelling [96]. The NGF-receptors are also a prognostic marker for oral squamous cell carcinomas with a pattern of invasion and recurrence [97].

Finally, cell-to-extracellular matrix interaction during basement membrane formation and branching morphogenesis is mediated by the integrin receptor Int-α6β1, while establishment of apical-basal polarity depends on Int-α3 and Int-β1 [98,99,100]. In vitro differentiation of salivary gland cell lines also requires Intα6β1 and its interaction with laminin1 [101]. In the adult tissue, Int-α6, Int-β1 and Int-β4 are expressed in a cohort of cells with multipotent potential [102]. Additionally, the autoimmune Sjögren’s syndrome is characterized by low levels of laminin-α1 [103].

A deeper knowledge of the molecular pathways involved in maturation, functionality and regeneration of salivary glands will greatly implement future studies aiming at restoring salivary function lost as a consequence of diseases or mechanical injuries.

## 6. Regenerative Medicine and Salivary Glands Stem Cells

Alteration of salivary gland functionality has deep implications in the life quality of the patient. Current therapeutic approaches are mainly symptomatic and constitute an attempt to attenuate discomfort. Local administration of synthetic substitutes of saliva only temporary alleviate distress, but rely on continuous administration and do not represent a definitive therapeutic solution. Therefore, several therapeutic fronts are currently in development to promote further regenerative therapies.

### Stem Cells Therapies

**Figure 4:**
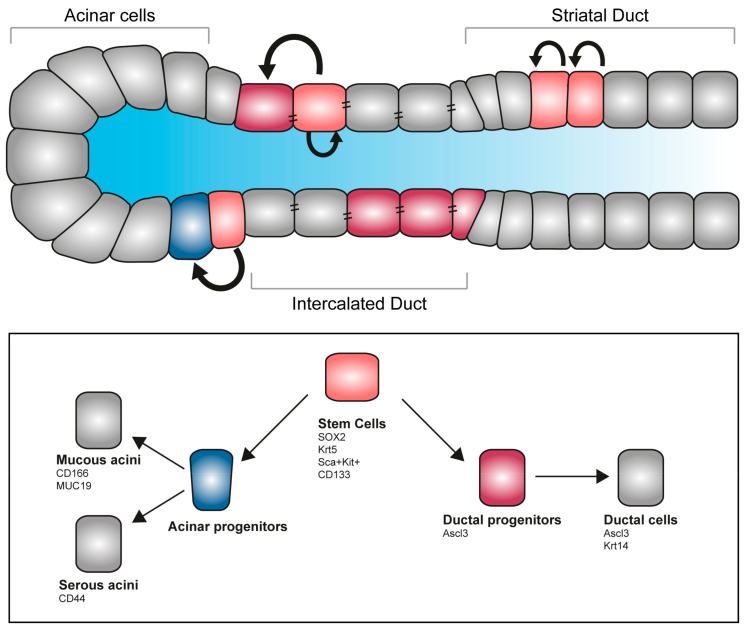
Stem cell pool and regeneration of salivary glands. Salivary glands contain several pools of undifferentiated progenitors able to self-renew, proliferate and give rise to differentiated acinar and ductal cells. Several markers allow the identification of stem cells and committed progenitors, allowing fate tracking and molecular discrimination.

Tissue degeneration and structural alteration are the main causes for salivary gland loss of function. Recent developments in regenerative therapy prompt the mobilization of endogenous stem cells, with promising results for the reconstitution of the damaged organ. Genetic tracing in animal models identified a cohort of progenitors in the major salivary glands of the adult. Long-lasting labelling revealed that, upon damage, acini can only give rise to new acinar cells, but not to ductal cells, suggesting the existence of an intrinsic fate-commitment program [104,105]. Specifically, a subset of the acinar population labelled with the transcription factor SOX2 has been reported to be able to differentiate into MUC19-expressing acinar cells and are responsible for acinar replenishment upon salivary gland irradiation [106] (Figure 4). As all major salivary glands of the adult possess a SOX2 population, it might be possible to exploit this population for regenerative purposes [107]. Other approaches are based on an in vitro system for stem cells expansion—culture of SMG-derived stem cells into salispheres allows amplification of the initial pool via self-renewal, while differentiation can be induced in vitro with functionally committed cells selectively producing mucin or amylase [108]. This population of stem cells can successfully be transplanted in vivo and ameliorates the effect of radiation exposure [109]. In the developing organ, a population of Kit-expressing cells localizes in the peripheral epithelial end-bud cells of the SMG. Kit signalling, together with the FGFr2b, directly influences the Krt14-expressing pool of distal progenitors, while in parallel preserving the Krt5^+^ epithelial progenitors and inducing survival of the neuronal niche [27]. Genetic tracings indicate that the Krt5 population remains as undifferentiated population on the adult gland, and mainly localizes in the SMG duct. Additionally, a dormant population of mesenchymal progenitor cells is present in healthy adult salivary glands. This Sca^+^/Kit^+^ population proliferates locally in response to injuries and has the potential to replenish the lost tissue forming both acini and ducti [110,111].

The transcription factor ASCL3 is expressed in a subset of ductal cells in the adult salivary glands. Lineage tracing experiments showed that they can give rise to both ductal and acinar cells [105,111]. Interestingly, ablation of the Ascl3^+^-population results in glands of smaller size, where a subpopulation of Krt5-progenitors still remains [112,113]. These results suggest that the salivary glands host more than one population of undifferentiated progenitors with the potential of compensate each other loss in case of selective depletion.

A drawback in the potential of therapeutic usage of stem cells for regenerative medicine is their ability to convert into cancer stem cells. Progenitors and cancer-initiating cells share several markers (such as SOX2, SOX9, SOX10 and MYC) [114,115,116,117]. On the other hand, the high variance of cancer types developed in the salivary glands indicate that tumours might originate from different types of cells at various stages of differentiation, most likely following heterogeneous tumorigenic programs [1,58].

The full restorative potential of salivary glands stem cells and its applicability remain to be further analysed, and studies on salivary glands reconstruction might be used as a platform to shed light on multiple biological and therapeutic aspects of epithelial regeneration.

## 7. Perspective and Future Directions

Several new technological settings have been recently developed to study the cellular and molecular dynamics governing salivary gland homeostasis and maintenance. Organotypic cultures together with live-imaging and in vivo tracing are cutting edge technologies that can mimic salivary glands dynamics and physiology. Likewise, the development of innovative platform modelling the complexity of salivary glands environment, provides the ideal substrate to establish novel, more efficient therapeutic approaches.

### 7.1. Biomimetic Models and Organ Cultures

Bi-dimensional tissue culture are limited by the lack of cell-to-cell and cell-to-extracellular matrix interaction. The influence of the surrounding microenvironment is recently gathering attention for its essential role in coordinating cell renewal, proliferation and differentiation. Similarly, in the context of disease modelling, taking into consideration an altered microenvironment is paramount to picture the complexity of crosstalk that sustain the disease (i.e., tumour microenvironment, inflammation, induced trans-differentiation).

Technological advances in 3D tissue culture allowed the development of functional, artificial salivary gland organs starting from patients’ cells. Cells seeded into a scaffold populate the bioengineered template, reproducing the structural complexity needed for biological, pharmacological and clinical studies [118,119]. To date, a major limitation is that primary cells display limited growth and finite life-span, which hampers the applicability of artificial organ transplants. Novel approaches are based on the usage of cell lines instead of primary cells, with promising preliminary results. Nevertheless, to date no cell line fully recapitulate endogenous salivary gland cells and therapeutic applicability is also delayed by their intrinsic tumorigenic potential [120,121,122,123].

### 7.2. Live Imaging of Functional Glands

Multi-coloured reporter lines are now available to study single cell dynamics in the context of the native organ environment [124,125]. This technology coupled with advances in the imaging field, allow precise definition of the single molecular elements controlling salivary glands functionality. A coloured cassette can be used to track fate definition of a specific population, determining the factors relevant for cell-type specification and tissue homeostasis [126,127]. Understanding the dynamics of the molecular changes in physiological or experimental conditions is essential to progress our knowledge of salivary gland biology. Additionally, disease modelling can be developed on a transgenic background and changes visualized over time in a functional and descriptive manner. Selection of cells based on timely expression of the transgene can then be coupled with molecular analyses and ad-hoc pathway activation. In contrast with previous analyses, tissue live imaging provides a method to observe single cells in their native environment, with minimal manipulation from the operator and, therefore, preserving unaltered the complexity of crosstalk between cells and their surrounding environmental cues.

### 7.3. Microfluidic Chambers

To provide a platform for functional studies, microfluidic chambers represent a novel approach for mechanical, pharmacological and biological characterization. The device contains several chambers where primary cells are exposed to a controlled composition of medium. These cells retain many characteristics of the original organ, including the ability to secrete and react to cytokines, growth factors and pharmacological compounds. Connection in between chambers can be finely regulated by liquid flow passage, whose composition varies depending on the modelling application. With the microfluidic model, cells adhesion, molecular and mechanical changes can be studied in a fine-regulated environment, dramatically improving screening efficiency and reducing the usage of animal models. Importantly, this system also allows rapid drug screening and novel therapeutic studies in a setting that strongly mimic the original tissue [128,129].

## 8. Conclusions

Salivary glands, therefore, represent a major player in the maintenance of oral homeostasis and their study might shed light in more general disorders such as cancer, inflammation and healing upon mechanical traumas. Overall, their accessibility and heterogeneous histology provide an ideal structure to improve our understanding of tissue remodelling and interaction between cells and surrounding microenvironment. In addition to studies on the molecular control of the exocrine function, salivary glands can be used as a platform to investigate the physiology of epithelial tissue, the dynamic of the stem cell niche and basic developmental processes. Thus, future studies on salivary glands might be impactful to a variety of subjects and application in biomedicine.

## Figures and Tables

**Table 1:** Salivary gland tumours. Salivary gland tumours are heterogeneous and can derive from different type of cells in the tissue. Presented here are the most common malignancies affecting the salivary glands.

Type	Origin	Most Common Location	Metastatic	5-Year Survival Rate	15-Year Survival Rate	Molecular Targets and Pathway Activation
**Mucoepidermoid Carcinoma**	Excretory stem cells	PG	Yes, regional lymphnodes	22–86%	--	Notch Hes1 CREB EGFR P53 Krt5
**Adenoid Cystic Carcinoma**	Intercalated stem cells	minor SG	Yes, lungs	89%	40%	Notch DNAmet TGF-β c-Kit Myb p63
**Acinic Cell Carcinoma**	Intercalated stem cells	PG		76%	55%	
**Polymorphous Adenocarcinoma**	Intercalated ductal cells	Minor SG	Rarely Perineural lymphnodes	--	--	p63
**Squamous Cell Carcinoma**	Excretory stem cells	PG, SMG	Neck region	--	--	
**Non-Hodgkin lymphoma**	Infiltrating immune cells	PG		--	--	
**Pleomorphic adenoma**	Intercalated stem cells	PG	no	--	--	

## Abbreviations

PG	Parotid gland
SL	Sublingual gland
SMG	Submandibular gland
EGF	Epidermal growth factor
FGF	Fibroblast growth factor
NGF	Nerve growth factor
TGF- α	Transforming growth factor- α
GCT	Granular convoluted tubules
PSG	Parasympathetic submandibular ganglion
Krt	Keratin
ER	Endoplasmatic reticulum
AQP5	Aquaporin 5
SNAP	Soluble NSF attachment proteins
SNAREs	SNAP REceptor
cAMP	Cyclic adenosine monophosphate
CREB	cAMP-responsive element binding
CRTC1	CREB Regulated Transcription Coactivator 1
MAML2	Mastermind-like 2
pSS	Primary Sjögren’s syndrome
TNF	Tumor necrosis factor
BAFF	B-cell activating factor
APRIL	A proliferation-inducing ligand
HIV	Human immunodeficiency viruses
GDNF	Glial cell line-derived neurotrophic factor
Int	Integrin
SOX	SRY-related HMG-box
MUC	Mucin

## References

[1] Amano O., Mizobe K., Bando Y., Sakiyama K. (2012). Anatomy and histology of rodent and human major salivary glands: Overview of the Japan salivary gland society-sponsored workshop. Acta Histochem. Cytochem..

[2] Dobrosielski-Vergona K. (1993). Biology of the Salivary Glands.

[3] Korsrud F.R., Brandtzaeg P. (1980). Quantitative immunohistochemistry of immunoglobulin- and J-chain-producing cells in human parotid and submandibular salivary glands. Immunology.

[4] Smith D.J., Taubman M.A., King W.F. (1987). Immunological features of minor salivary gland saliva. J. Clin. Immunol..

[5] Treuting P.M., Dintzis S.M., Frevert C.W., Liggitt D., Liggitt H.D., Montine K.S. (2012). Comparative Anatomy and Histology: A Mouse and Human Atlas (Expert Consult).

[6] Kondo Y., Nakamoto T., Jaramillo Y., Choi S., Catalan M.A., Melvin J.E. (2015). Functional differences in the acinar cells of the murine major salivary glands. J. Dent. Res..

[7] Matsuo R. (2000). Role of saliva in the maintenance of taste sensitivity. Crit. Rev. Oral Biol. Med..

[8] Ono Minagi H., Sarper S.E., Kurosaka H., Kuremoto K.I., Taniuchi I., Sakai T., Yamashiro T. (2017). Runx1 mediates the development of the granular convoluted tubules in the submandibular glands. PLoS ONE.

[9] Mori M., Sumitomo S., Shrestha P., Tanaka S., Takai Y., Shikimori M. (2008). Multifunctional roles of growth factors or biologically active peptides in salivary glands and saliva. Oral Med. Pathol..

[10] Jiménez-Rojo L., Granchi Z., Graf D., Mitsiadis T.A. (2012). Stem Cell Fate Determination during Development and Regeneration of Ectodermal Organs. Front. Physiol..

[11] Gervais E.M., Sequeira S.J., Wang W., Abraham S., Kim J.H., Leonard D., DeSantis K.A., Larsen M. (2016). Par-1b is required for morphogenesis and differentiation of myoepithelial cells during salivary gland development. Organogenesis.

[12] Chatzeli L., Gaete M., Tucker A.S. (2017). Fgf10 and Sox9 are essential for the establishment of distal progenitor cells during mouse salivary gland development. Development.

[13] Carlson B.M. (2008). Human Embryology and Developmental Biology E-Book.

[14] Carlson B.M. (2009). Human Embryology and Developmental Biology.

[15] Pagella P., Jiménez-Rojo L., Mitsiadis T.A. (2014). Roles of innervation in developing and regenerating orofacial tissues. Cell Mol. Life Sci..

[16] Coughlin M.D. (1975). Early development of parasympathetic nerves in the mouse submandibular gland. Dev. Biol..

[17] Patel V.N., Rebustini I.T., Hoffman M.P. (2006). Salivary gland branching morphogenesis. Differentiation.

[18] Walker J.L., Menko A.S., Khalil S., Rebustini I., Hoffman M.P., Kreidberg J.A., Kukuruzinska M.A. (2008). Diverse roles of E-cadherin in the morphogenesis of the submandibular gland: Insights into the formation of acinar and ductal structures. Dev. Dyn..

[19] Kwon H.R., Nelson D.A., DeSantis K.A., Morrissey J.M., Larsen M. (2017). Endothelial cell regulation of salivary gland epithelial patterning. Development.

[20] Lee M.G., Ohana E., Park H.W., Yang D., Muallem S. (2012). Molecular mechanism of pancreatic and salivary gland fluid and HCO3 secretion. Physiol. Rev..

[21] Iaizzo P.A., He B. (2013). Introduction to Neurophysiology. Neural Eng..

[22] Lundberg A. (1958). Electrophysiology of salivary glands. Physiol Rev..

[23] Alm P. (1973). Adrenergic and cholinergic nerves of bovine, guinea pig and hamster salivary glands. A light and electron microscopic study. Z. Zellforsch. Mikrosk. Anat..

[24] Garrett J.R. (1972). Neuro-Effector Sites in Salivary Glands. Oral Physiology.

[25] Garret J.R., Kidd A. (1976). Effects of autonomic nerve stimulation on submandibular acini and saliva in cats [proceedings]. J. Physiol..

[26] Patel V.N., Hoffman M.P. (2014). Salivary gland development: A template for regeneration. Semin. Cell Dev. Biol..

[27] Knox S.M., Lombaert I.M.A., Reed X., Vitale-Cross L., Gutkind J.S., Hoffman M.P. (2010). Parasympathetic innervation maintains epithelial progenitor cells during salivary organogenesis. Science.

[28] Makita T., Sucov H.M., Gariepy C.E., Yanagisawa M., Ginty D.D. (2008). Endothelins are vascular-derived axonal guidance cues for developing sympathetic neurons. Nature.

[29] Ventimiglia M.S., Rodriguez M.R., Morales V.P., Elverdin J.C., Perazzo J.C., Castañ M.M., Davio C.A., Vatta M.S., Bianciotti L.G. (2011). Endothelins participate in the central and peripheral regulation of submandibular gland secretion in the rat. Am. J. Physiol. Regul. Integr. Comp. Physiol..

[30] Glebova N.O., Ginty D.D. (2004). Heterogeneous requirement of NGF for sympathetic target innervation in vivo. J. Neurosci..

[31] Levi-Montalcini R., Angeletti P.U. (1968). Nerve growth factor. Physiol. Rev..

[32] Murphy R.A., Saide J.D., Blanchard M.H., Young M. (1977). Nerve growth factor in mouse serum and saliva: Role of the submandibular gland. Proc. Natl. Acad. Sci. USA.

[33] Garrett J.R., Kidd A. (1993). The innervation of salivary glands as revealed by morphological methods. Microsc. Res. Tech..

[34] Proctor G.B., Carpenter G.H. (2007). Regulation of salivary gland function by autonomic nerves. Auton. Neurosci..

[35] Nanci A. (2012). Ten Cate’s Oral Histology.

[36] Delporte C., Bryla A., Perret J. (2016). Aquaporins in Salivary Glands: From Basic Research to Clinical Applications. Int. J. Mol. Sci..

[37] Takuma T., Tagaya M., Ichida T. (1997). Evidence for the putative docking/fusion complex of exocytosis in parotid acinar cells. FEBS Lett..

[38] Fujita-Yoshigaki J., Dohke Y., Hara-Yokoyama M., Kamata Y., Kozaki S., Furuyama S., Sugiya H. (1996). Vesicle-associated membrane protein 2 is essential for cAMP-regulated exocytosis in rat parotid acinar cells. The inhibition of cAMP-dependent amylase release by botulinum neurotoxin B. J. Biol. Chem..

[39] Garrett J.R., Thulin A. (1975). Changes in parotid acinar cells accompanying salivary secretion in rats on sympathetic or parasympathetic nerve stimulation. Cell Tissue Res..

[40] Segawa A., Terakawa S., Yamashina S., Hopkins C.R. (1991). Exocytosis in living salivary glands: Direct visualization by video-enhanced microscopy and confocal laser microscopy. Eur. J. Cell Biol..

[41] Roukema P.A., Oderkerk C.H., Salkinoga-Salonen M.S. (1976). The murine sublingual and submandibular mucins. Their isolation and characterization. Biochim. Biophys. Acta.

[42] Kim S.K., Nasjleti C.E., Han S.S. (1972). The secretion processes in mucous and serous secretory cells of the rat sublingual gland. J. Ultrastruct. Res..

[43] Garrett J.R. (1987). The proper role of nerves in salivary secretion: A review. J. Dent. Res..

[44] Vreugdenhil A.P., Nieuw Amerongen A.V., De Lange G.L., Roukema P.A. (1982). Localization of amylase and mucins in the major salivary glands of the mouse. Histochem. J..

[45] Ibrahim H.R., Thomas U., Pellegrini A. (2001). A helix-loop-helix peptide at the upper lip of the active site cleft of lysozyme confers potent antimicrobial activity with membrane permeabilization action. J. Biol. Chem..

[46] Laible N.J., Germaine G.R. (1985). Bactericidal activity of human lysozyme, muramidase-inactive lysozyme, and cationic polypeptides against Streptococcus sanguis and Streptococcus faecalis: Inhibition by chitin oligosaccharides. Infect. Immun..

[47] Sava G., Benetti A., Ceschia V., Pacor S. (1989). Lysozyme and cancer: Role of exogenous lysozyme as anticancer agent (review). Anticancer Res..

[48] Sun H., Chen Y., Zou X., Li Q., Li H., Shu Y., Li X., Li W., Han L., Ge C. (2016). Salivary Secretory Immunoglobulin (SIgA) and Lysozyme in Malignant Tumor Patients. Biomed. Res. Int..

[49] Noble R.E. (2000). Salivary alpha-amylase and lysozyme levels: A non-invasive technique for measuring parotid vs submandibular/sublingual gland activity. J. Oral Sci..

[50] Veerman E.C., Van den Keybus P.A., Vissink A., Nieuw Amerongen A.V. (1996). Human glandular salivas: Their separate collection and analysis. Eur. J. Oral Sci..

[51] Garrett J.R., Emmelin N. (1979). Activities of salivary myoepithelial cells: A review. Med. Biol..

[52] Emmelin N., Garrett J.R., Gjörstrup P. (1977). Supporting effects of myoepithelial cells in submandibular glands of dogs when acting against increased intraluminal pressure. J. Physiol..

[53] Senthilkumar B., Mahabob M.N. (2012). Mucocele: An unusual presentation of the minor salivary gland lesion. J. Pharm. Bioallied Sci..

[54] Pinkston J.A., Cole P. (1999). Incidence rates of salivary gland tumors: Results from a population-based study. Otolaryngol. Head Neck Surg..

[55] Stenner M., Klussmann J.P. (2009). Current update on established and novel biomarkers in salivary gland carcinoma pathology and the molecular pathways involved. Eur. Arch. Otorhinolaryngol..

[56] Alvi S., Chudek D., Limaiem F. (2019). Cancer, Parotid. StatPearls.

[57] Yan K., Yesensky J., Hasina R., Agrawal N. (2018). Genomics of mucoepidermoid and adenoid cystic carcinomas. Laryngoscope Investig. Otolaryngol..

[58] Emmerson E., Knox S.M. (2018). Salivary gland stem cells: A review of development, regeneration and cancer. Genesis.

[59] Manvikar V., Ramulu S., Ravishanker S.T., Chakravarthy C. (2014). Squamous cell carcinoma of submandibular salivary gland: A rare case report. J. Oral Maxillofac. Pathol..

[60] Mendenhall W.M., Mendenhall C.M., Werning J.W., Malyapa R.S., Mendenhall N.P. (2008). Salivary gland pleomorphic adenoma. Am. J. Clin. Oncol..

[61] Chen Z., Chen J., Gu Y., Hu C., Li J.L., Lin S., Shen H., Cao C., Gao R., Li J. (2014). Aberrantly activated AREG-EGFR signaling is required for the growth and survival of CRTC1-MAML2 fusion-positive mucoepidermoid carcinoma cells. Oncogene.

[62] Chen J., Li J.-L., Chen Z., Griffin J.D., Wu L. (2015). Gene expression profiling analysis of CRTC1-MAML2 fusion oncogene-induced transcriptional program in human mucoepidermoid carcinoma cells. BMC Cancer.

[63] Tonon G., Modi S., Wu L., Kubo A., Coxon A.B., Komiya T., O’Neil K., Stover K., El-Naggar A., Griffin J.D. (2003). t(11;19)(q21;p13) translocation in mucoepidermoid carcinoma creates a novel fusion product that disrupts a Notch signaling pathway. Nat. Genet..

[64] Behboudi A., Enlund F., Winnes M., Andrén Y., Nordkvist A., Leivo I., Flaberg E., Szekely L., Mäkitie A. (2006). Molecular classification of mucoepidermoid carcinomas-prognostic significance of the MECT1-MAML2 fusion oncogene. Genes Chromosomes Cancer.

[65] Stephens P.J., Davies H.R., Mitani Y., Van Loo P., Shlien A., Tarpey P.S., Papaemmanuil E., Cheverton A., Bignell G.R., Butler A.P. (2013). Whole exome sequencing of adenoid cystic carcinoma. J. Clin. Investig..

[66] Rettig E.M., Talbot C.C., Sausen M., Jones S., Bishop J.A., Wood L.D., Tokheim C., Niknafs N., Karchin R., Fertig E.J. (2016). Whole-Genome Sequencing of Salivary Gland Adenoid Cystic Carcinoma. Cancer Prev. Res..

[67] Chen W., Cao G., Yuan X., Zhang X., Zhang Q., Zhu Y., Dong Z., Zhang S. (2015). Notch-1 knockdown suppresses proliferation, migration and metastasis of salivary adenoid cystic carcinoma cells. J. Transl. Med..

[68] Qu J., Song M., Xie J., Huang X.Y., Hu X.M., Gan R.H., Zhao Y., Lin L.S., Chen J., Lin X. (2016). Notch2 signaling contributes to cell growth, invasion, and migration in salivary adenoid cystic carcinoma. Mol. Cell Biochem..

[69] Groom J., Kalled S.L., Cutler A.H., Olson C., Woodcock S.A., Schneider P., Tschopp J., Cachero T.G., Batten M., Wheway J. (2002). Association of BAFF/BLyS overexpression and altered B cell differentiation with Sjögren’s syndrome. J. Clin. Investig..

[70] Mackay F., Browning J.L. (2002). BAFF: A fundamental survival factor for B cells. Nat. Rev. Immunol..

[71] He B., Chadburn A., Jou E., Schattner E.J., Knowles D.M., Cerutti A. (2004). Lymphoma B cells evade apoptosis through the TNF family members BAFF/BLyS and APRIL. J. Immunol..

[72] Mariette X., Seror R., Quartuccio L., Baron G., Salvin S., Fabris M., Desmoulins F., Nocturne G., Ravaud P., De Vita S. (2015). Efficacy and safety of belimumab in primary Sjögren’s syndrome: Results of the BELISS open-label phase II study. Ann. Rheum. Dis..

[73] De Vita S., Quartuccio L., Salvin S., Picco L., Scott C.A., Rupolo M., Fabris M. (2014). Sequential therapy with belimumab followed by rituximab in Sjögren’s syndrome associated with B-cell lymphoproliferation and overexpression of BAFF: Evidence for long-term efficacy. Clin. Exp. Rheumatol..

[74] Steinfeld S.D., Tant L., Burmester G.R., Teoh N.K., Wegener W.A., Goldenberg D.M., Pradier O. (2006). Epratuzumab (humanised anti-CD22 antibody) in primary Sjögren’s syndrome: An open-label phase I/II study. Arthritis Res. Ther..

[75] Dass S., Bowman S.J., Vital E.M., Ikeda K., Pease C.T., Hamburger J., Richards A., Rauz S., Emery P. (2008). Reduction of fatigue in Sjögren syndrome with rituximab: Results of a randomised, double-blind, placebo-controlled pilot study. Ann. Rheum. Dis..

[76] Devauchelle-Pensec V., Mariette X., Jousse-Joulin S., Berthelot J.M., Perdriger A., Puéchal X., Le Guern V., Sibilia J., Gottenberg J.E., Chiche L. (2014). Treatment of primary Sjögren syndrome with rituximab: A randomized trial. Ann. Intern. Med..

[77] Vissink A., Mitchell J.B., Baum B.J., Limesand K.H., Jensen S.B., Fox P.C., Elting L.S., Langendijk J.A., Coppes R.P., Reyland M.E. (2010). Clinical management of salivary gland hypofunction and xerostomia in head-and-neck cancer patients: Successes and barriers. Int. J. Radiat. Oncol. Biol. Phys..

[78] Burlage F.R., Coppes R.P., Meertens H., Stokman M.A., Vissink A. (2001). Parotid and submandibular/sublingual salivary flow during high dose radiotherapy. Radiother. Oncol..

[79] Pinna R., Campus G., Cumbo E., Mura I., Milia E. (2015). Xerostomia induced by radiotherapy: An overview of the physiopathology, clinical evidence, and management of the oral damage. Ther. Clin. Risk Manag..

[80] Visvanathan V., Nix P. (2010). Managing the patient presenting with xerostomia: A review. Int. J. Clin. Pract..

[81] Aframian D.J., Mizrahi B., Granot I., Domb A.J. (2010). Evaluation of a mucoadhesive lipid-based bioerodable tablet compared with Biotène mouthwash for dry mouth relief—A pilot study. Quintessence Int..

[82] Villa A., Connell C.L., Abati S. (2015). Diagnosis and management of xerostomia and hyposalivation. Ther. Clin. Risk Manag..

[83] Wilson K.F., Meier J.D., Ward P.D. (2014). Salivary gland disorders. Am. Fam. Phys..

[84] Lombaert I.M.A., Brunsting J.F., Wierenga P.K., Kampinga H.H., De Haan G., Coppes R.P. (2008). Keratinocyte growth factor prevents radiation damage to salivary glands by expansion of the stem/progenitor pool. Stem Cells.

[85] Häärä O., Fujimori S., Schmidt-Ullrich R., Hartmann C., Thesleff I., Mikkola M.L. (2011). Ectodysplasin and Wnt pathways are required for salivary gland branching morphogenesis. Development.

[86] Hai B., Yang Z., Millar S.E., Choi Y.S., Taketo M.M., Nagy A., Liu F. (2010). Wnt/β-catenin signaling regulates postnatal development and regeneration of the salivary gland. Stem Cells Dev..

[87] Jaskoll T., Leo T., Witcher D., Ormestad M., Astorga J., Bringas P., Carlsson P., Melnick M. (2004). Sonic hedgehog signaling plays an essential role during embryonic salivary gland epithelial branching morphogenesis. Dev. Dyn..

[88] Hai B., Qin L., Yang Z., Zhao Q., Shangguan L., Ti X., Zhao Y., Kim S., Rangaraj D., Liu F. (2014). Transient activation of hedgehog pathway rescued irradiation-induced hyposalivation by preserving salivary stem/progenitor cells and parasympathetic innervation. Clin. Cancer Res..

[89] Haberman A.S., Isaac D.D., Andrew D.J. (2003). Specification of cell fates within the salivary gland primordium. Dev. Biol..

[90] Mitsiadis T.A., Henrique D., Thesleff I., Lendahl U. (1997). Mouse Serrate-1 (Jagged-1): Expression in the developing tooth is regulated by epithelial-mesenchymal interactions and fibroblast growth factor-4. Development.

[91] Dang H., Lin A.L., Zhang B., Zhang H.-M., Katz M.S., Yeh C.-K. (2009). Role for Notch signaling in salivary acinar cell growth and differentiation. Dev. Dyn..

[92] Jhappan C., Gallahan D., Stahle C., Chu E., Smith G.H., Merlino G., Callahan R. (1996). Expression of an activated Notch-related int-3 transgene interferes with cell differentiation and induces neoplastic transformation in mammary and salivary glands. Genes Dev..

[93] Rossi J., Luukko K., Poteryaev D., Laurikainen A., Sun Y.F., Laakso T., Eerikäinen S., Tuominen R., Lakso M., Rauvala H. (1999). Retarded growth and deficits in the enteric and parasympathetic nervous system in mice lacking GFR alpha2, a functional neurturin receptor. Neuron.

[94] Cohen S. (2004). Origins of growth factors: NGF and EGF. Ann. N. Y. Acad. Sci..

[95] Ghasemlou N., Krol K.M., Macdonald D.R., Kawaja M.D. (2004). Comparison of target innervation by sympathetic axons in adult wild type and heterozygous mice for nerve growth factor or its receptor trkA. J. Pineal Res..

[96] Schenck K., Schreurs O., Hayashi K., Helgeland K. (2017). The Role of Nerve Growth Factor (NGF) and Its Precursor Forms in Oral Wound Healing. Int. J. Mol. Sci..

[97] Søland T.M., Brusevold I.J., Koppang H.S., Schenck K., Bryne M. (2008). Nerve growth factor receptor (p75 NTR) and pattern of invasion predict poor prognosis in oral squamous cell carcinoma. Histopathology.

[98] Kadoya Y., Kadoya K., Durbeej M., Holmvall K., Sorokin L., Ekblom P. (1995). Antibodies against domain E3 of laminin-1 and integrin alpha 6 subunit perturb branching epithelial morphogenesis of submandibular gland, but by different modes. J. Cell Biol..

[99] Menko A.S., Kreidberg J.A., Ryan T.T., Van Bockstaele E., Kukuruzinska M.A. (2001). Loss of alpha3beta1 integrin function results in an altered differentiation program in the mouse submandibular gland. Dev. Dyn..

[100] Rebustini I.T., Patel V.N., Stewart J.S., Layvey A., Georges-Labouesse E., Miner J.H., Hoffman M.P. (2007). Laminin alpha5 is necessary for submandibular gland epithelial morphogenesis and influences FGFR expression through beta1 integrin signaling. Dev. Biol..

[101] Hecht D., Jung D., Prabhu V.V., Munson P.J., Hoffman M.P., Kleinman H.K. (2002). Metallothionein promotes laminin-1-induced acinar differentiation in vitro and reduces tumor growth in vivo. Cancer Res..

[102] Sato A., Okumura K., Matsumoto S., Hattori K., Hattori S., Shinohara M., Endo F. (2007). Isolation, tissue localization, and cellular characterization of progenitors derived from adult human salivary glands. Cloning Stem Cells.

[103] Laine M., Virtanen I., Salo T., Konttinen Y.T. (2004). Segment-specific but pathologic laminin isoform profiles in human labial salivary glands of patients with Sjogren’s syndrome. Arthritis Rheum..

[104] Lemercier C., To R.Q., Swanson B.J., Lyons G.E., Konieczny S.F. (1997). Mist1: A novel basic helix-loop-helix transcription factor exhibits a developmentally regulated expression pattern. Dev. Biol..

[105] Yoshida S., Ohbo K., Takakura A., Takebayashi H., Okada T., Abe K., Nabeshima Y. (2001). Sgn1, a basic helix-loop-helix transcription factor delineates the salivary gland duct cell lineage in mice. Dev. Biol..

[106] Arnold K., Sarkar A., Yram M.A., Polo J.M., Bronson R., Sengupta S., Seandel M., Geijsen N., Hochedlinger K. (2011). Sox2(+) adult stem and progenitor cells are important for tissue regeneration and survival of mice. Cell Stem Cell.

[107] Emmerson E., May A.J., Berthoin L., Cruz-Pacheco N., Nathan S., Mattingly A.J., Chang J.L., Ryan W.R., Tward A.D., Knox S.M. (2018). Salivary glands regenerate after radiation injury through SOX2-mediated secretory cell replacement. EMBO Mol. Med..

[108] Feng J., van der Zwaag M., Stokman M.A., van Os R., Coppes R.P. (2009). Isolation and characterization of human salivary gland cells for stem cell transplantation to reduce radiation-induced hyposalivation. Radiother. Oncol..

[109] Lombaert I.M.A., Brunsting J.F., Wierenga P.K., Faber H., Stokman M.A., Kok T., Visser W.H., Kampinga H.H., de Haan G., Coppes R.P. (2008). Rescue of salivary gland function after stem cell transplantation in irradiated glands. PLoS ONE.

[110] Hisatomi Y., Okumura K., Nakamura K., Matsumoto S., Satoh A., Nagano K., Yamamoto T., Endo F. (2004). Flow cytometric isolation of endodermal progenitors from mouse salivary gland differentiate into hepatic and pancreatic lineages. Hepatology.

[111] Bullard T., Koek L., Roztocil E., Kingsley P.D., Mirels L., Ovitt C.E. (2008). Ascl3 expression marks a progenitor population of both acinar and ductal cells in mouse salivary glands. Dev. Biol..

[112] Arany S., Catalán M.A., Roztocil E., Ovitt C.E. (2011). Ascl3 knockout and cell ablation models reveal complexity of salivary gland maintenance and regeneration. Dev. Biol..

[113] Rugel-Stahl A., Elliott M.E., Ovitt C.E. (2012). Ascl3 marks adult progenitor cells of the mouse salivary gland. Stem Cell Res..

[114] Schröck A., Bode M., Göke F.J.M., Bareiss P.M., Schairer R., Wang H., Weichert W., Franzen A., Kirsten R., van Bremen T. (2014). Expression and role of the embryonic protein SOX2 in head and neck squamous cell carcinoma. Carcinogenesis.

[115] Matheu A., Collado M., Wise C., Manterola L., Cekaite L., Tye A.J., Canamero M., Bujanda L., Schedl A., Cheah K.S. (2012). Oncogenicity of the developmental transcription factor Sox9. Cancer Res..

[116] Ivanov S.V., Panaccione A., Nonaka D., Prasad M.L., Boyd K.L., Brown B., Guo Y., Sewell A., Yarbrough W.G. (2013). Diagnostic SOX10 gene signatures in salivary adenoid cystic and breast basal-like carcinomas. Br. J. Cancer.

[117] Schoenhals M., Kassambara A., De Vos J., Hose D., Moreaux J., Klein B. (2009). Embryonic stem cell markers expression in cancers. Biochem. Biophys. Res. Commun..

[118] Redman R.S. (2008). On approaches to the functional restoration of salivary glands damaged by radiation therapy for head and neck cancer, with a review of related aspects of salivary gland morphology and development. Biotech. Histochem..

[119] Nelson J., Manzella K., Baker O.J. (2013). Current cell models for bioengineering a salivary gland: A mini-review of emerging technologies. Oral Dis..

[120] Aframian D.J., Tran S.D., Cukierman E., Yamada K.M., Baum B.J. (2002). Absence of tight junction formation in an allogeneic graft cell line used for developing an engineered artificial salivary gland. Tissue Eng..

[121] Warner J.D., Peters C.G., Saunders R., Won J.H., Betzenhauser M.J., Gunning W.T., Yule D.I., Giovannucci D.R. (2008). Visualizing form and function in organotypic slices of the adult mouse parotid gland. Am. J. Physiol. Gastrointest. Liver. Physiol..

[122] Yanagawa T., Hayashi Y., Nagamine S., Yoshida H., Yura Y., Sato M. (1986). Generation of cells with phenotypes of both intercalated duct-type and myoepithelial cells in human parotid gland adenocarcinoma clonal cells grown in athymic nude mice. Virchows Arch. B Cell Pathol..

[123] Shirasuna K., Sato M., Miyazaki T. (1981). A neoplastic epithelial duct cell line established from an irradiated human salivary gland. Cancer.

[124] Pontes-Quero S., Heredia L., Casquero-García V., Fernández-Chacón M., Luo W., Hermoso A., Bansal M., Garcia-Gonzalez I., Sanchez-Muñoz M.S., Perea J.R. (2017). Dual ifgMosaic: A Versatile Method for Multispectral and Combinatorial Mosaic Gene-Function Analysis. Cell.

[125] Snippert H.J., van der Flier L.G., Sato T., van Es J.H., van den Born M., Kroon-Veenboer C., Barker N., Klein A.M., van Rheenen J., Simons B.D. (2010). Intestinal crypt homeostasis results from neutral competition between symmetrically dividing Lgr5 stem cells. Cell.

[126] Soriano P. (1999). Generalized lacZ expression with the ROSA26 Cre reporter strain. Nat. Genet..

[127] Muzumdar M.D., Tasic B., Miyamichi K., Li L., Luo L. (2007). A global double-fluorescent Cre reporter mouse. Genesis.

[128] Mosier A.P., Peters S.B., Larsen M., Cady N.C. (2014). Microfluidic platform for the elastic characterization of mouse submandibular glands by atomic force microscopy. Biosensors.

[129] Kong J., Luo Y., Jin D., An F., Zhang W., Liu L., Li J., Fang S., Li X., Yang X. (2016). A novel microfluidic model can mimic organ-specific metastasis of circulating tumor cells. Oncotarget.

